# Selective Oxidation of Toluene to Benzaldehyde Using Co-ZIF Nano-Catalyst

**DOI:** 10.3390/ijms232112881

**Published:** 2022-10-25

**Authors:** Wei Long, Zhilong Chen, Yinfei Huang, Xinping Kang

**Affiliations:** 1 College of Chemistry, Guangdong University of Petrochemical Technology, Maoming 525000, China; 2 Guangdong Provincial Key Laboratory of Petrochemical Pollution Process and Control, Guangdong University of Petrochemical Technology, Maoming 525000, China; 3 College of Electromechanical Engineering, Guangdong University of Petrochemical Technology, Maoming 525000, China

**Keywords:** selective oxidation, benzaldehyde, nanometer-size catalyst, MOFs material

## Abstract

Nanometer-size Co-ZIF (zeolitic imidazolate frameworks) catalyst was prepared for selective oxidation of toluene to benzaldehyde under mild conditions. The typical characteristics of the metal-organic frameworks (MOFs) material were affirmed by the XRD, SEM, and TEM, the BET surface area of this catalyst was as high as 924.25 m^2^/g, and the diameter of particles was near 200 nm from TEM results. The Co metal was coated with 2-methyl glyoxaline, and the crystalline planes were relatively stable. The reaction temperatures, oxygen pressure, mass amount of N-hydroxyphthalimide (NHPI), and reaction time were discussed. The Co-ZIF catalyst gave the best result of 92.30% toluene conversion and 91.31% selectivity to benzaldehyde under 0.12 MPa and 313 K. The addition of a certain amount of NHPI and the smooth oxidate capacity of the catalyst were important factors in the high yield of benzaldehyde. This nanometer-size catalyst showed superior performance for recycling use in the oxidation of toluene. Finally, a possible reaction mechanism was proposed. This new nanometer-size Co-ZIF catalyst will be applied well in the selective oxidation of toluene to benzaldehyde.

## 1. Introduction

As the industry rapidly grows, volatile organic compounds (VOCs) are known as organic carbon-based chemicals that evaporate easily, causing serious human health and environmental pollution problems [1]. The relationship between many major environmental problems, such as global warming, photochemical ozone formation, stratospheric ozone depletion, and VOCs, is distinct and serious [2]. As significant air pollutants, VOCs are controlled at low concentrations when they are discharged from a chimney. However, the maximum emission concentration of VOCs is different in varied countries or regions [3]. Among the carbon-based VOCs, toluene, xylene, and benzene are found to be endocrine-disrupting chemicals, exerting severe toxicity to animals or humans [4]. They are the major source of very fine particulate matter or ozone that produces heavy, smoggy, unfriendly, harmful, and hazy weather [5]. Hence, it is essential to develop the technology level of VOCs transition.

Catalytic total oxidation has been considered the most appropriate method and economical route for VOCs removal, and many efforts have been made to design catalysts with suitable activity and selectivity [6,7,8]. Catalytic oxidation is an effective technology to completely convert VOCs into CO_2_ and H_2_O without any by-products at low temperatures [9]. The total oxidation of airborne VOCs into carbon dioxide and water at room temperature can be performed in the presence of a semiconductor catalyst [10]. However, the total oxidation of VOCs only can reduce pollution emissions and convert them directly into usable chemical material reuse wastes, which has great significance [11]. Selective oxidation products of VOCs are commonly used in various applications, including adhesives, paints, rubbers, leather tanning processes, and as chemical solvents to dissolve many organic substances [12]. Catalytic selective oxidation has been considered the most appropriate method for VOC removal, and many efforts have been made to design catalysts with suitable activity and selectivity [13,14,15].

The commercial catalysts for oxidation of VOCs can be classified into three categories: (1) supported noble metals [16,17]; (2) metal oxides or supported metals [18,19]; (3) mixtures of noble metals and metal oxides [20,21]. Noble metal catalysts generally show higher activity and selectivity to organic acid, transition metal oxides are one alternative to the noble metal-containing catalysts due to their resistance to halogens, low cost and high catalytic activity and selectivity to aldehyde [22]. As the high value chemicals and suitable chemical raw materials, aldehyde are very popular to chemical engineering researchers. Selective oxidation of VOCs is very suitable channel to reuse waste, but this process is difficult to unify considering the purification and separation of products [23]. As a typical example, selective oxidation of toluene to benzaldehyde have been received many attempts and reports from researchers in different areas of the world [24,25,26,27].

Many new catalysts have been invented and verified for this selective oxidation reaction, and many catalytic reaction mechanisms were proposed [28]. Xie et al. [29] prepared three-dimensionally ordered macroporous supported gold-palladium alloy catalyst (Au-Pd/3DOM Co_3_O_4_) which performed highly active and suitable stable for toluene oxidation, stronger noble metal-3DOM Co_3_O_4_ interaction was the key factor, but the catalyst cost is too high. New MnO_2_ sample (MnO_2_-P) exhibited the higher content of oxygen vacancy, which could enhance reducibility and mobility of lattice oxygen, so higher activity and best catalytic performance were obtained in selective oxidation reaction of toluene [30]. Yuan et al. [31] collected much experimental results with a wide range of conditions from low to high temperatures, normal atmospheric to high pressures, very lean to pyrolysis conditions and developed kinetic model of selective oxidation of toluene. Nevertheless, the ideal catalyst, which can show the properties as high product selectivity, suitable recycling use capacity, low cost, mild reaction conditions, and fast reaction speed has not been found. Hence, there is still space for the development of a catalyst that has higher activity and better selective to benzaldehyde in the oxidation of toluene.

Recently, MOF materials have shown a great advantage for CO_2_ capture and separation [32], and Ni-MOF can exhibit great catalytic capacity in heterogeneous catalytic reactions such as CO_2_ hydrogenation [33]. Usman et al. [34] proposed that MOF/g-C_3_N_4_ composites will be important photocatalysts for prospective applications in energy harvesting and controlling environmental pollution. Shafiq et al. [35] found the imidazole framework-95 (ZIF-95) MOF could be dispersed in polysulfone polymer to form mixed matrix membranes, so gas separation performance and selectivity of polysulfone membrane were enhanced greatly. Hence, Large surface area, tunable pore size and window, and tailored functionalities are the great advantages of MOFs, which can be used for the efficient catalyst.

**Scheme 1:**
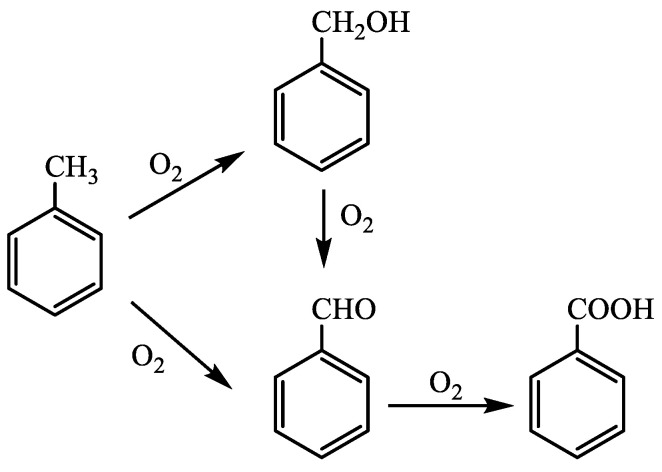
The catalytic oxidation process of toluene [36].

In the present study, the selective catalytic oxidation process of toluene was shown by Scheme 1 [36]. Toluene can be oxidated to benzyl alcohol, and it is oxidated easily to benzaldehyde. The final oxidation product is benzoic acid. Recently, metal-organic frameworks (MOFs) materials have been used widely in the field of catalysis, and it shows obvious feature as high surface area, complex porous structure, and obvious cavity coordination [36]. As the stable catalyst, Co-ZIF is prepared and used in this oxidation process of toluene. Considering the control of selective oxidation of reactant, NHPI as the reaction initiator is added to the solution. The optimized reaction conditions are found, and better catalytic performance results are confirmed, so the advantages of this MOF catalyst are true and reliable.

## 2. Results

### Characterization of Catalysts

**Figure 1:**
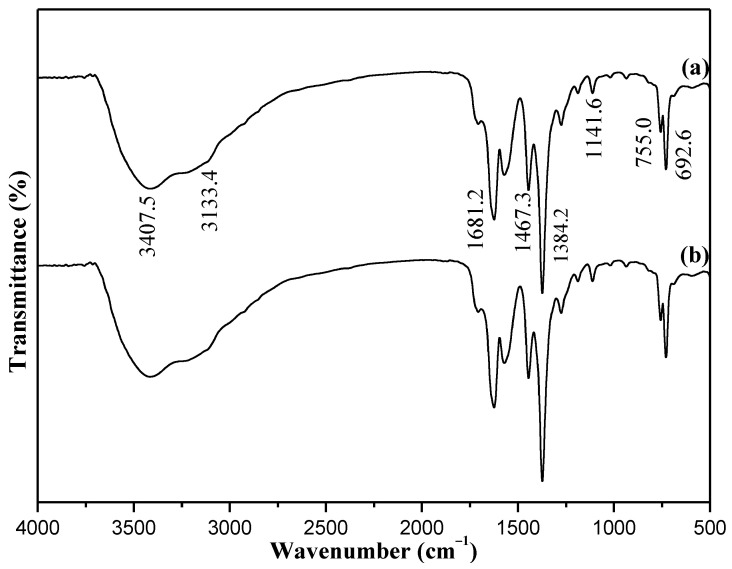
FT-IR spectrum of Co-ZIF catalyst. (**a**) fresh, (**b**) after reaction.

FT-IR spectra of catalyst samples are shown in Figure 1, and there are some characteristic adsorption peaks of MOFs material in the curves. The obvious peak at 3407.5 and 3133.4 cm^−1^ are ascribed to the stretching vibration of the O-H bond of the crystal water molecule and the C-H bond of MOFs material, respectively. Many small peaks near 1467.3 cm^−^^1^ and 1141.6 cm^−^^1^ belong to the stretching vibration of the C-N bond in the MOFs material (Figure 1a). The acute peak at 1483.2 cm^−^^1^ belongs to the bending vibration of the C-N bond in the MOFs material. The distinct peaks near 1681.2 cm^−^^1^ are ascribed to the stretching vibration of the N-H bond in the 2-methyl glyoxaline molecule. Two overlap peaks at 755.0 cm^−1^ and 692.6 cm^−1^ are attributed to the bending vibration of the N-H bond.

The characteristic adsorption peak at 1846.2 cm^−^^1^ is the important signal of the coordinate bond [37]. There are no peaks near 1846.2 cm^−^^1^, indicating the coordinate bond of Co-N has been established successfully. Obviously, there are no apparent changes or weaknesses in the curve of the used catalyst (Figure 1b), so the Co-ZIF catalyst exhibits suitable stability.

**Figure 2:**
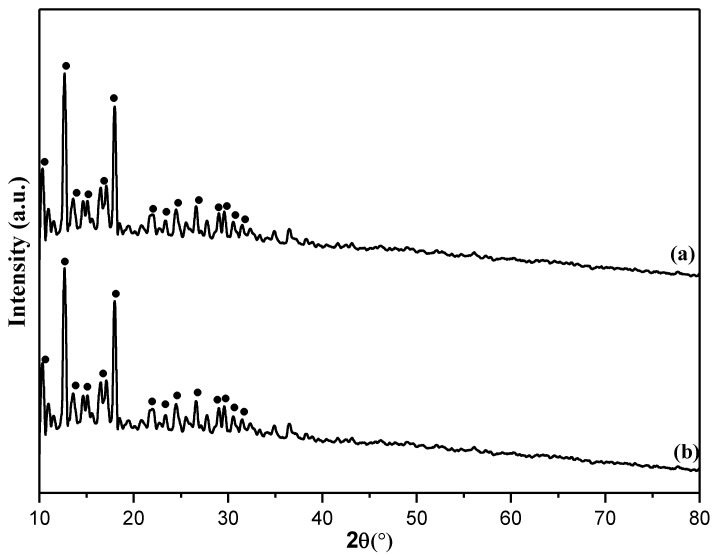
X-ray diffraction (XRD) patterns of Co-ZIF catalyst. (**a**) fresh, (**b**) after reaction.

The XRD spectra of the samples (Co-ZIF catalyst, fresh and after reaction) are shown in Figure 2. The characteristic diffraction peaks at 2θ = 10.4°, 12.7°, 13.3°, 14.8°, 16.5°, 18.0°, 22.1°, 24.5°, 25.5°, 26.7°, 29.5°, 30.6°, 31.6°, and 32.5° are ascribed to crystalline planes of Co-ZIF material, which are different with crystalline planes of Co_3_O_4_ particles [38]. As the typical MOFs material, the crystallinity of the Co-ZIF catalyst was low, so the intensity of many characteristic diffraction peaks was low (Figure 2a). Two apparent peaks at 2θ = 12.7° and 18.0° might are ascribed to miller indices of Co_3_O_4_ [211] and Co_3_O_4_ [111], respectively. This strange phenomenon might be elucidated because there was trace cobalt oxide (Co_3_O_4_) in the catalyst sample, but there are no other evident diffraction peaks of Co_3_O_4_ particles, so it is not pure cobalt oxide, and the MOFs constituent is affirmed.

We can see similar XRD signals for the used catalyst sample in Figure 2b, so the crystalline planes are relatively stable, and the stability of this MOF material maybe is suitable. These strange phenomena in the XRD spectra might be due to the forceful coordinate effect in the MOFs material. The average particle size of Co-ZIF species was approx. 18.4 nm, which was calculated from the XRD data based on the Scherrer equation.

**Figure 3:**
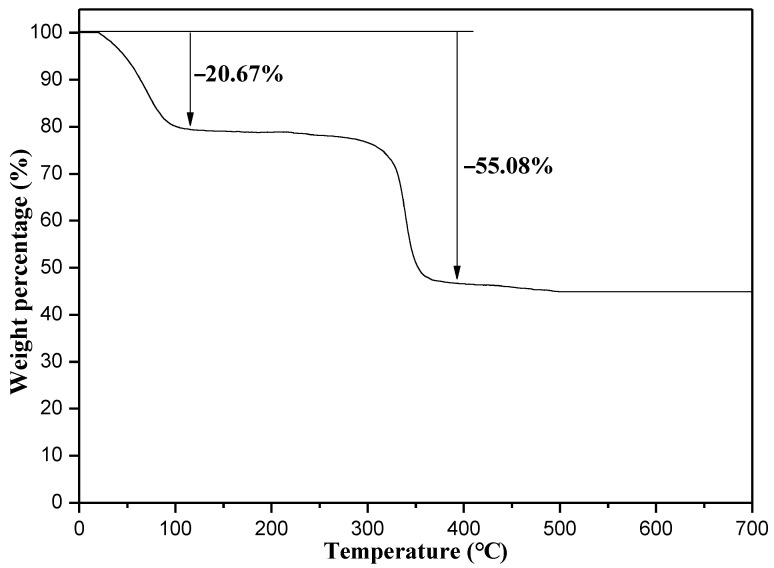
TG curves of Co-ZIF catalyst.

TG curves of the Co-ZIF catalyst are shown in Figure 3, and there are two obvious weightlessness stages in the curve. The first weightlessness rate is 20.67% since the temperature reaches 120 °C due to the loss of crystal water in catalyst powder. The reason might be ascribed to the drying temperature being low in the preparation processing. The second weightlessness rate is 55.08% since the temperature reaches 380 °C due to the deformation or decomposition of MOFs material. This weightlessness occurs from 300 °C to 380 °C, so the stability of the Co-ZIF catalyst is poor, and it only is used in the reaction under 300 °C. The curve shows very flat from 400 °C to 700 °C might indicate that the microstructures of the catalyst have collapsed, and cobaltous oxide and carbide is the major constituent. These results are consistent with the typical characteristics of MOFs materials, and the reaction temperature should be controlled below 300 °C in the oxidizing reaction of toluene.

**Figure 4:**
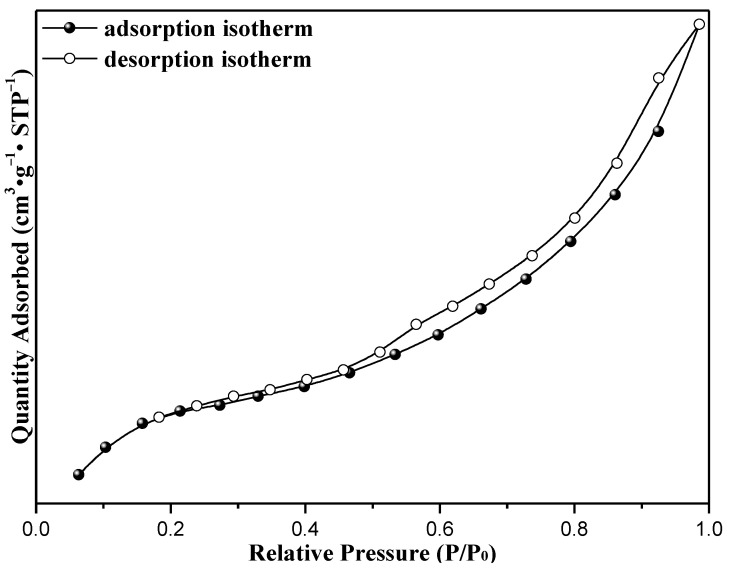
N_2_ adsorption-desorption isotherms of the Co-ZIF catalyst.

The high specific surface area and lots of homogeneous pores are the typical features of MOFs materials, so the BET characterization can be used for the textural properties of this catalyst. N_2_ adsorption-desorption isotherms of the Co-ZIF catalyst are displayed in Figure 4, and the pore size distributions of the Co-ZIF catalyst are shown in Figure S1.

Obviously, the N_2_ isothermal adsorption-desorption curve is of type II according to the Brunauer–Deming–Deming–Teller classification, which means the strong interaction between adsorbate and surface. The hugeous multi-layer adsorption exists when the vapor pressure reaches saturation. The hystersis loop is of type H3 according to the IUPAC classification, which means the catalyst has the feature of irregular pores. The initial isotherm curvatures of the prepared catalyst sample are assigned to micro- and mesopores, while the ascending curvature of the plateau in the high relative pressure range (P/P_0_ = 0.85–1.00) is inherent to macropores [39]. This strange phenomenon might be ascribed to the slit holes by cumulus of MOF flaky structures, and adsorption saturation does not occur at higher pressure.

The BET surface area of this catalyst is 924.25 m^2^·g^−1^, the pore volume data are 0.429 cm^3^·g^−1^, and there is a little solvent blocking the pore in the preparation process, and it does not affect the reaction. The pore diameter distribution is relatively concentrated in Figure S1, and the homogeneous pores of this MOF catalyst are affirmed, and this structure might be beneficial for the reaction. However, a wide pore size distribution ranging from ~2.0 nm to ~22.0 nm in Figure S1 further confirms the microporous-dominated structure of this catalyst. The obtained BJH adsorption average pore width of this catalyst is 4.0 nm. In contrast, the wider range of pore size distribution, BJH average pore width, and higher adsorbed volume indicate partial mesopores in this catalyst [40]. The presence of simultaneous micro-, meso-, and macropores in this catalyst indicate the formation of the hierarchical porous structure. Such a combination of the assessable porous structure is advantageous for capturing reactant molecules through the micropores, while the meso- and macropores play an effective role in developing contact probability between the reactant molecule and active centers of the catalyst.

**Figure 5:**
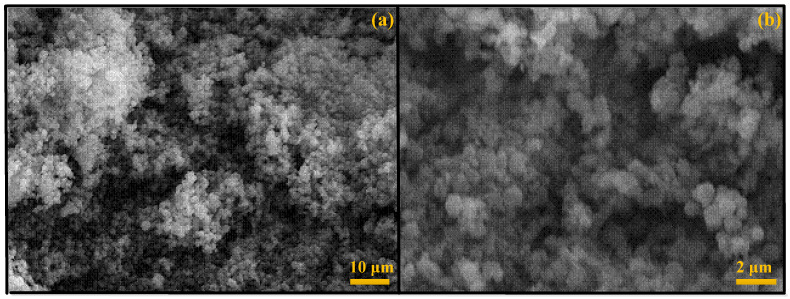
SEM images of the Co-ZIF catalyst. (**a**) fresh catalyst at magnification of 2000; (**b**) fresh catalyst at magnification of 10,000.

The SEM images of the Co-ZIF catalyst are displayed in Figure 5. The microparticle shape of the catalyst is clear and has apparent coral characteristics in Figure 5a. After further magnification, the particles of the MOFs catalyst are piled, and there are many pores on the surface. The diameter of major particles is less than 1 μm, and many dark channels might be the overlap of particles. This result is consistent with the BET results, and the typical characteristics of the MOFs material are confirmed.

**Figure 6:**
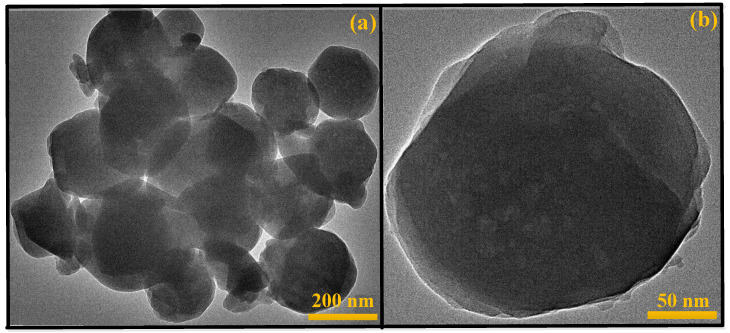
TEM images of the Co-ZIF catalyst. (**a**) fresh catalyst at magnification of 100,000; (**b**) fresh catalyst at magnification of 400,000.

The TEM images of the Co-ZIF catalyst are displayed in Figure 6, and there are many circular dark balls shown in Figure 6a. There is a certain gap among the ball’s site, and the arrangement model is not uniform. In addition, we can measure the particle size in Figure 6b and the diameter near 200 nm, so the nano-scale catalyst has been confirmed. There are many rough convex objects and dark ditches on the surface of particles, so the Co metal might be coated with 2-methyl glyoxaline tightly.

**Figure 7:**
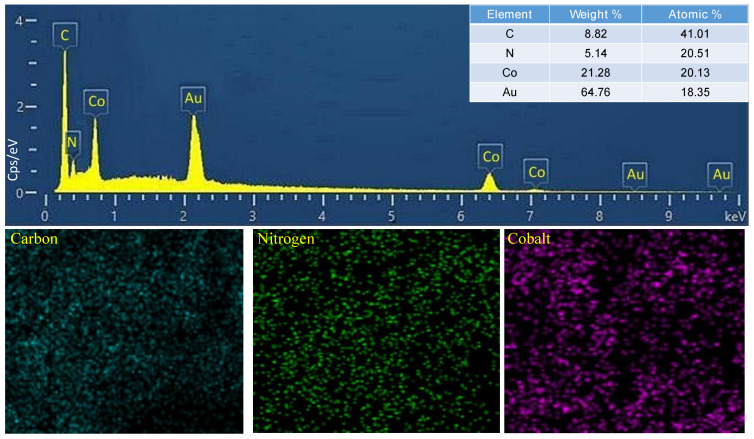
EDS spectra of Co-ZIF catalyst and elemental mapping.

The EDS spectra of the Co-ZIF catalyst and elemental mapping are shown in Figure 7. The elemental peaks correspond to C, N, Co, and Au. The major contribution of the C, N, and Co EDS peaks suggests the well surface element distribution. The obvious peaks of Au in Figure 7 are not evitable due to the conventional preparation method used. The uniform distribution of the major elements as C, N, and Co can reflect the successful combination of MOFs materials.

## 3. Discussion

### 3.1. Catalytic Performance

**Figure 8:**
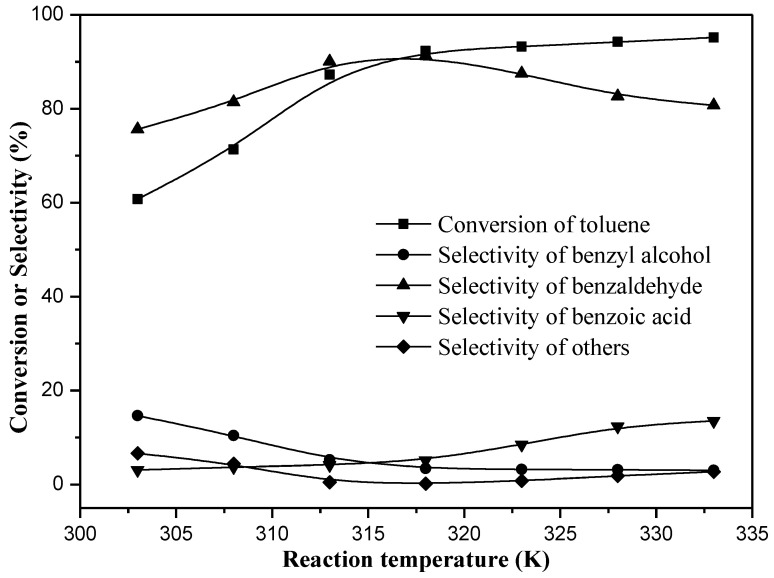
Effect of reaction temperature. Reaction conditions 0.20 g Co-ZIF, 0.500 mmol toluene, 20 mL HFIP, 0.004 g NHPI, 0.12 MPa O_2_ and 240 min.

The reaction temperature is the important factor for the selective oxidation of toluene, and the effects of the reaction temperature are shown in Figure 8. It is clearly found that reaction temperature has a great effect on this catalytic performance, and the selectivity of products is different at different temperatures. The conversion of toluene increases distinctly from 60.72% to 92.30% since the reaction temperature rises from 303 K to 318 K, and the selectivity to benzaldehyde increases from 75.64% to 91.31%. However, this increasing trend slows down since the reaction temperature exceeds 318 K, and the selectivity to benzaldehyde decreases clearly. The selectivity to benzoic acid increases rapidly, so the higher reaction temperature may promote the transformation of benzaldehyde to benzoic acid. The optimum temperature is 313 K, and the maximum yield of benzaldehyde reached 84.28%.

The effects of O_2_ pressure are listed in Table 1, and the effect of the O_2_ pressure is not as obvious as the reaction temperature. The conversion of toluene and the selectivity to benzoic acid increase with the increment of O_2_ pressure, but the selectivity to benzaldehyde reach the maximum value (91.31%) when the O_2_ pressure is 0.12 MPa. The selectivity to benzyl alcohol is significantly reduced distinctly from 8.14% to 2.67%, so the higher O_2_ pressure may benefit the deep oxidation of toluene. The selectivity to others reaches the lowest value when the O_2_ pressure is 0.12 MPa, so the inevitable side reaction maybe can not be neglected in this reaction system.

The effects of toluene concentrations are listed in Table 2. We can identify that the conversion capacity of the catalyst is limited. When the toluene concentration was 25.0 mmol/L, the conversion of toluene was not as high as 100%. The conversion of toluene cut down with the increment of toluene concentrations, so the appropriate initialized toluene concentrations were an important factor in developing the yield of major products. The selectivity of benzyl alcohol increased clearly with the increment of the initialized toluene concentrations, which indicated that the oxidation reaction was insufficient. The maximum selectivity of benzaldehyde was 91.31% when the initialized toluene concentration was 25.0 mmol/L, and the corresponding yield of benzaldehyde was as high as 84.28%.

The effects of the mass of the catalyst are listed in Table S1. The conversion of toluene increased clearly with the increment of the catalyst. The selectivity of benzaldehyde reached maximum since the mass of the catalyst was 0.20 g. Too much catalyst can develop the selectivity of benzoic acid, so the content of benzaldehyde was obviously reduced. Hence, the suitable mass of the catalyst in this reaction was 0.20 g.

**Figure 9:**
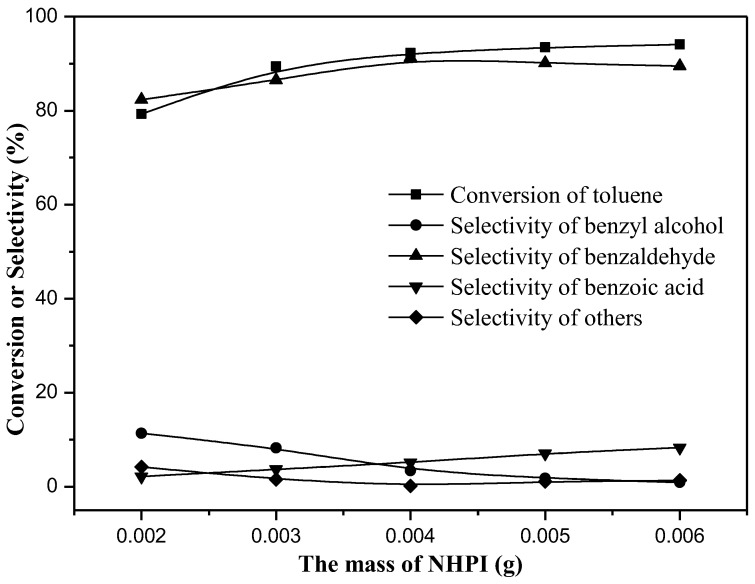
Effect of the mass amount of NHPI. Reaction conditions 0.20 g Co-ZIF, 0.500 mmol toluene, 20 mL HFIP, 0.12 MPa O_2_, 313 K and 240 min.

The effects of the mass of NHPI are shown in Figure 9. It is obvious that the addition of NHPI can develop the catalytic activity of the catalyst, which is proved by the variation tendency of selectivity of benzoic acid. The conversion of toluene is as low as 6.74%, and benzoic acid is the major product when the NHPI is not added to this reaction system. The catalytic activity of the catalyst was low without the NHPI. As the role of initiator, the addition of NHPI is necessary. The suitable mass of NHPI in this reaction was 0.004 g, and the selectivity of benzaldehyde reached the maximum.

Although NHPI and toluene can be oxidized by nanocatalysts, the further oxidation of benzyl alcohol and benzaldehyde maybe occur at the same time. Too much NHPI also is not suitable in Figure 9. The selectivity of benzoic acid increases rapidly, and the selectivity of benzyl alcohol decreases distinctly with the increment of NHPI mass. This strange phenomenon might be ascribed to the activity of an intermediate compound that was affected by NHPI.

**Figure 10:**
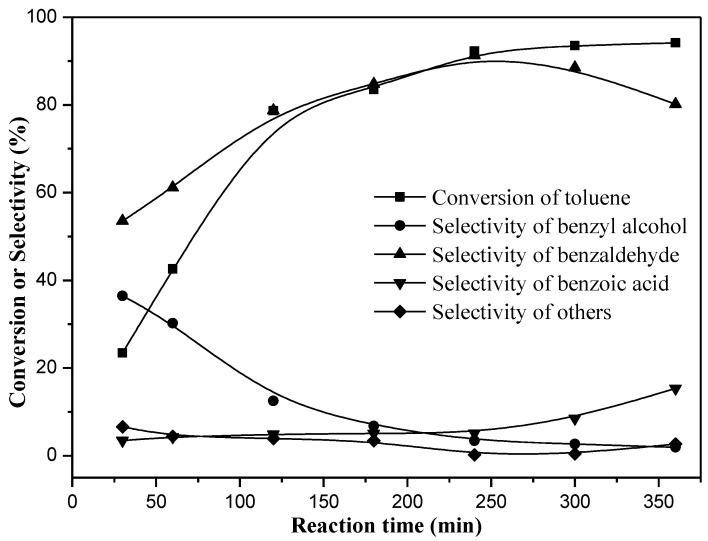
Effect of the reaction time. Reaction conditions 0.20 g Co-ZIF, 0.500 mmol toluene, 20 mL HFIP, 0.004 g NHPI, 0.12 MPa O_2_ and 313 K.

The effects of reaction time are displayed in Figure 10. It is obvious that the conversion of toluene increases rapidly with the prolonged reaction time. The conversion of toluene can reach 90% since the reaction time is 240 min, but the variation tendency of the conversion of toluene is not obvious since the reaction time is greater than 240 min. So the catalytic reaction reached a dynamic equilibrium since the reaction time was 240 min.

For the selectivity of products, the reaction time might be an important factor. We saw the selectivity of benzaldehyde cut down since the reaction time was more than 240 min. The selectivity to benzoic acid increased as the reaction time went on. We also found that the selectivity of benzyl alcohol cut down clearly with the increment of reaction time. Toluene was oxidized to benzyl alcohol, and the benzyl alcohol was oxidized to benzaldehyde or benzoic acid. Hence, proper oxidation is beneficial to the formation of benzaldehyde and the suitable reaction time for selective oxidation of toluene over a nanometer-size Co-ZIF catalyst is 240 min under 313 K and 0.12 MPa.

Many nano catalysts containing Mn or Co were prepared for the oxidation of toluene, and permanganate was used for the oxidation of toluene as early as 1975 [41]. The ability of permanganate to abstract a hydrogen atom is rationalized on the basis of the strong O-H bond formed on H· addition to permanganate, which is a key step of C-H bond oxidations. A comparison of the oxidation of toluene catalyzed by various catalysts is listed in Table 3. Different Mn or Co metal catalysts are better for the oxidation of toluene, and the major products are different. A complete oxidation process also can be performed to generate carbon dioxide, which is significant for the removal of toluene. The catalytic activity and advantage of the Co-ZIF catalyst are clear in Table 3, and this oxidation by the Co-ZIF catalyst belonged to selective oxidation. Benzaldehyde was the major product, and the yield (84.3%) was higher than other catalysts.

**Figure 11:**
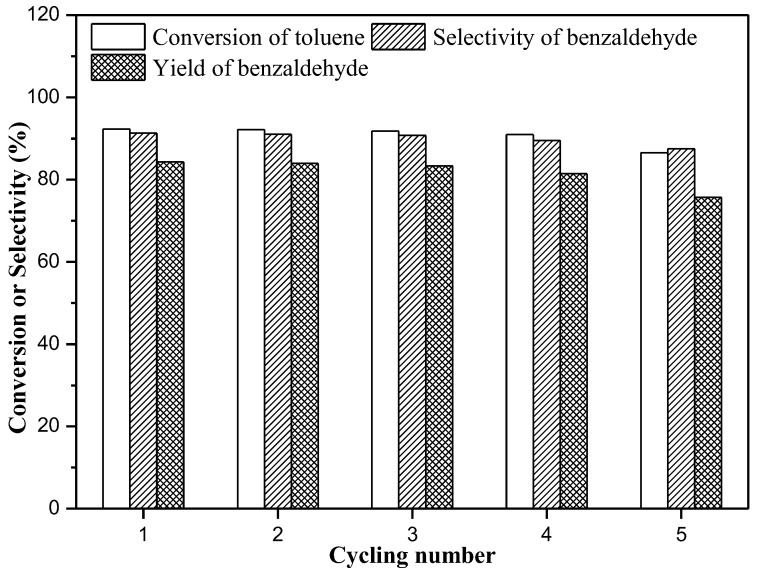
The recycling of Co-ZIF catalyst. Reaction conditions 0.20 g Co-ZIF, 0.500 mmol toluene, 20 mL HFIP, 0.004 g NHPI, 0.12 MPa O_2_, 313 K and 240 min.

The results of the reusability of the Co-ZIF catalyst are shown in Figure 11. Typically, the catalysts are separated by filtration, washed and dried in vacuum, and then used in the next run. NHPI is the important initiator in this reaction system, which is added in every new cycle of the process (0.004 g). The catalytic performance decreases a little until the fourth recycle. Nevertheless, the toluene conversion decreases to 86.53%, and the selectivity to benzaldehyde decreases to 87.46% for the fifth time. Hence, the catalytic activity of Co-ZIF is very stable, and it is suitable for recycling use in the oxidation of toluene.

Additionally, the effects of solvent over the Co-ZIF catalyst are shown in Supplementary Table S2. It can be seen that the polar solvent is helpful for the selective oxidation of toluene, and the high polar solvent of HFIP gives the best catalytic performance of 92.30% conversion and 91.31% selectivity to benzaldehyde. As the strong polar solvent, HFIP is beneficial to the oxidation of the C-H bond, and the dehydrogenation reaction is easier to proceed by the action of free radicals. So benzyl alcohol is easier to produce. Another important factor is that the hydrogen bond between benzaldehyde and HFIP can inhibit the further oxidation of benzaldehyde, so the yield of benzaldehyde can be improved clearly.

### 3.2. Probable Catalytic Mechanism

**Figure 12:**
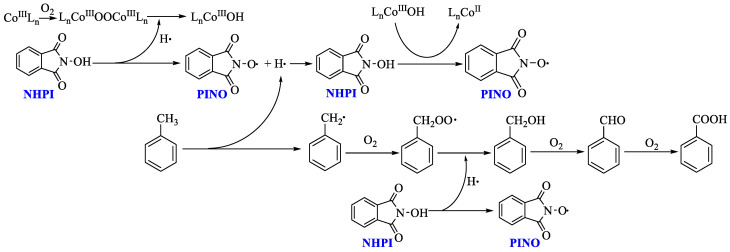
The proposed mechanism of selective oxidation of toluene by Co-ZIF catalyst.

Based on the results from this work and the literature [38,49], a possible reaction path for the oxidation of toluene over the Co-ZIF catalyst was proposed and shown in Figure 12. The free radical reaction mechanism maybe is a popular reasonable explanation. PINO can be generated by the deprotonation of NHPI. It also is related to the participation of a catalyst. Co^III^L_n_ represents the elementary unit of the Co-ZIF catalyst, and it is oxidized to the strong oxidation active intermediate as L_n_Co^III^OOCo^III^L_n_ by oxygen [47]. PINO can be generated by the deproton reaction under this strong oxidation active intermediate. The dehydrogenation of toluene is the first step of the main chain reaction, so the attractive proton capacity of PINO is the decisive influence in these steps, which indicates that NHPI can be restored. Another way is the oxidation of L_n_Co^III^OH, which can transform NHPI to PINO.

Toluene radical can be generated from toluene by the strong proton attraction of PINO, and it is oxidized by oxygen into the peroxide intermediate (benzyl peroxy radical). Then, benzyl alcohol can be formed by the transformation of benzyl peroxy radical with a free proton from NHPI [50]. In the step of chain termination, benzyl alcohol is oxidized to benzaldehyde and further oxidized to benzoic acid. The intermediate products of toluene radical, PINO and benzyl peroxy radical were testified by GC-MS. Considering the boiling point of PINO is much higher than the others, vacuum distillation or rectification can be used for the further separation of products and the PINO radical. Hence, the yield of benzaldehyde is affected by the mass amount of NHPI, but the catalytic activity of NHPI is affected by the Co-ZIF catalysts.

## 4. Materials and Methods

### 4.1. Materials

Co(NO_3_)_2_·6H_2_O, 2-methyl glyoxaline, dimethylformamide (DMF), toluene, benzyl alcohol, benzaldehyde were purchase from Shanghai Macklin Biochemical Co., Ltd. (Shanghai, China) N-hydroxyl phthalimide (NHPI), benzoic acid, 1,1,1,3,3,3-hexafluoroisopropanol (HFIP), ethanol, and methanol were analytical grade and purchased from Sinopharm Chemical Reagent Co., Ltd (Tianjin, China). As an internal standard in the meteorological chromatographic analysis procedure, acetophenone were chromatographically pure and purchased from Shanghai Macklin Biochemical Co., Ltd (Shanghai, China). O_2_ (99.99%) was provided by Maoming Deyuan Gas Company (Maoming, China).

### 4.2. Catalyst Preparation

The hydrothermal synthesis method [51] was chosen for the preparation of catalysts. Firstly, 1.478 g of 2-methyl glyoxaline was dispersed in 20 mL of DMF solution with stirred continuously for 1 h. Then, a certain amount of Co(NO_3_)_2_·6H_2_O crystal was added to this mixture solution and stirred continuously for 2 h. All liquid reactants were transferred into the crystallization autoclave and stayed in the cabinet dryer at 140 °C for 24 h. After being cooled to room temperature, the product powder was washed with distilled water and methanol several times. The purple crystalline powder was dried in air overnight at 80 °C and labeled as Co-ZIF.

### 4.3. Catalyst Characterization

Fourier transform infrared (FT-IR) spectra of the catalysts were recorded on a PerkinElmer spectrum One (C) spectrometer in the wave number range of 500–4000 cm^−1^. TG/DTG curves of the catalyst were recorded by Netzsch 210C thermogravimetric using air as purge gas (40 mL/min) over a temperature range of 35–800 °C with a heating rate of 10 °C/min. Powder X-ray diffraction (XRD) patterns of catalysts were recorded by the Bruker D/max 2515TC diffractometer device with Cu Kα radiation (λ = 1.535 Å). The scan range was 5–90°with with a scanning rate of 2°/min, and the tube voltage and current were 45 kV and 35 mA, respectively. The specific surface area, pore volume, and pore size distribution of the catalysts were analyzed by the nitrogen adsorption-desorption on a Quantachrome NOVA-2301D automated gas sorption system. Specific surface areas were calculated by Brunauer–Emmett–Teller (BET), and pore size distributions were calculated by Barrett-Joyner-Halenda (BJH) methods. The morphologies of the catalysts were observed by scanning electron microscope (SEM, JEOL 6510 ED) device. The microstructure of the catalyst was observed by transmission electron microscopy (TEM, TecnaiG350 SE) device, the working voltage at less than 200 kV, and the catalyst powder was deposited on a copper grid.

### 4.4. Catalytic Test Method

The catalytic oxidation process of toluene was performed in a 50 mL Teflon-lined stainless steel autoclave with a magnetic stirrer at 600 rpm. Typically, 20 mL HFIP, 0.500 mmol toluene, and 0.04 g NHPI were mixed into the quartz lining, 0.20 g catalyst powder was added into the mixture solution, and the quartz lining was transferred into the autoclave. The reactor was sealed and purged with O_2_ to exclude air three times, and then it was pressurized to 0.12 MPa with O_2_ under continuous stirring after the designed temperature was reached.

After a certain reaction time, the catalysts were separated by filtration carefully, and the gas phase products and the liquid phase products were analyzed by GC-MS. The gas phase products include oxygen, carbon dioxide, carbon monoxide, benzene, etc. The liquid phase products and contents of the reactants were analyzed by gas chromatography (Agilent Technologies, 8960B) equipped with a DB-7501 capillary column (diameter 0.60 mm, length 28 m) and a flame ionization detector (FID) using acetophenone as the internal standard substance (sample was diluted by ethanol). The major product was benzaldehyde, and by-products were benzyl alcohol, benzoic acid, benzyl benzoate, biphenyl, etc.

Considering the gas phase products are rare and the liquid phase products are important, all the concerned products contain an aromatic nucleus. We can ignore the carbon proportion. The toluene conversion (*w*) and product selectivity (*S*_i_) were calculated by the following equations [47].

(1) $$w\;(\%)=\frac{Mol{\left(toluene\right)}_{in}-Mol{\left(toluene\right)}_{out}}{Mol{\left(toluene\right)}_{in}}\times 100$$

(2) $$S_{i}(\%)=\frac{Mol_{i}}{Mol_{reacted\cdot toluene}}\times 100\;$$

## 5. Conclusions

In summary, nanometer-size Co-ZIF catalyst was prepared and characterized [37,38]. The catalyst presents a suitable catalytic performance in the selective oxidation of toluene to benzaldehyde under mild conditions. The typical characteristics of the MOFs material were affirmed by the SEM and TEM. The diameter of particles is near 200 nm, and the BET surface area of this catalyst is as high as 924.25 m^2^/g. The Co metal is coated with 2-methyl glyoxaline, and the crystalline planes are relatively stable. The addition of a certain amount of NHPI and the smooth oxidate capacity of the catalyst is in favor of a high yield of benzaldehyde. Under the optimized reaction conditions, the best catalytic performance of 92.30% conversion of toluene and the selectivity to benzaldehyde is up to 91.31%. This nanometer-size catalyst showed superior performance for recycling use in the selective oxidation of toluene. The possible reaction path is proposed based on GC-MS and the works of other researchers.

## Figures and Tables

**Table 1:** Effects of the O_2_ pressure.

O_2_ Pressure (MPa)	Conversion (%)	Selectivity (%)			
		Benzyl Alcohol	Benzaldehyde	Benzoic Acid	Others
0.06	79.35	8.14	82.17	2.17	7.52
0.08	86.32	6.42	86.58	3.24	3.76
0.10	90.11	4.13	89.32	4.39	2.16
0.12	92.30	3.42	91.31	5.13	0.14
0.14	92.56	2.98	89.14	6.57	1.31
0.16	92.88	2.67	86.34	8.46	2.53

Reaction conditions 0.20 g Co-ZIF, 0.500 mmol toluene, 20 mL HFIP, 0.004 g NHPI, 313 K and 240 min.

**Table 2:** Effects of the initialized toluene molar.

Toluene Concentration (mmol/L)	Conversion (%)	Selectivity (%)			
		Benzyl Alcohol	Benzaldehyde	Benzoic Acid	Others
6.25	100.00	1.03	72.69	22.46	3.82
12.5	100.00	2.37	83.79	12.37	1.47
25.0	92.30	3.42	91.31	5.13	0.14
37.5	73.48	12.49	80.49	4.16	2.86
50.0	52.87	23.17	70.34	3.38	3.11

Reaction conditions 0.20 g Co-ZIF, 20 mL HFIP, 0.004 g NHPI, 0.12 MPa O_2_, 313 K and 240 min.

**Table 3:** Comparison of oxidation of toluene catalyzed by various catalysts.

Catalysts	*w* (%)	Major Product	*S*_i_ (%)	Years	Ref.
Co(OAc)_2_·4H_2_O	91.0	Benzaldehyde	90.0	2002	[42]
Mn_0.67_-Cu_0.33_	99.0	Carbon dioxide	-	2004	[43]
Cobalt tetraphenylporphyrin	8.9	Benzaldehyde and benzyl alcohol	60.0	2005	[44]
Fe_2_O_3_-Mn_2_O_3_	100.0	Carbon monoxide and carbon dioxide	-	2009	[45]
CoO_x_/SiO_2_	91.2	Benzaldehyde	68.8	2019	[46]
ZIF-67-24	87.9	Benzaldehyde	66.9	2020	[47]
Au/γ-Al_2_O_3_	99.0	Carbon dioxide	-	2022	[48]
Co-ZIF	92.3	Benzaldehyde	91.3	in this work	

## Data Availability

Data are contained within the article or Supplementary Materials. The data presented in this study are available in Supplementary Materials.

## Supplementary Materials

The following supporting information can be downloaded at: https://www.mdpi.com/article/10.3390/ijms232112881/s1.
