# Supplementary material for: Selective Oxidation of Toluene to Benzaldehyde Using Co-ZIF Nano-Catalyst
Source: Int J Mol Sci. 2022 Oct 25;23(21):12881. doi: 10.3390/ijms232112881 (PMC9656501; doi:10.3390/ijms232112881)
Supplement: Supplementary file 1 [file ijms-23-12881-s001.zip › ijms-1925462-supplementary.pdf]

Article: Supplementary Materials

Selective Oxidation of Toluene to Benzaldehyde Using Co-ZIF Nano-Catalyst

Wei Long [1,2,*], Zhilong Chen [1], Yinfei Huang [3] and Xinping Kang [1]

[1] College of Chemistry, Guangdong University of Petrochemical Technology, Maoming 525000, China

[2] Guangdong Provincial Key Laboratory of Petrochemical Pollution Process and Control, Guangdong University of Petrochemical Technology, Maoming 525000, China

[3] College of Electromechanical Engineering, Guangdong University of Petrochemical Technology, Maoming 525000, China

* Correspondence: longwei@gdupt.edu.cn. Tel.: +86-0668-2337639.

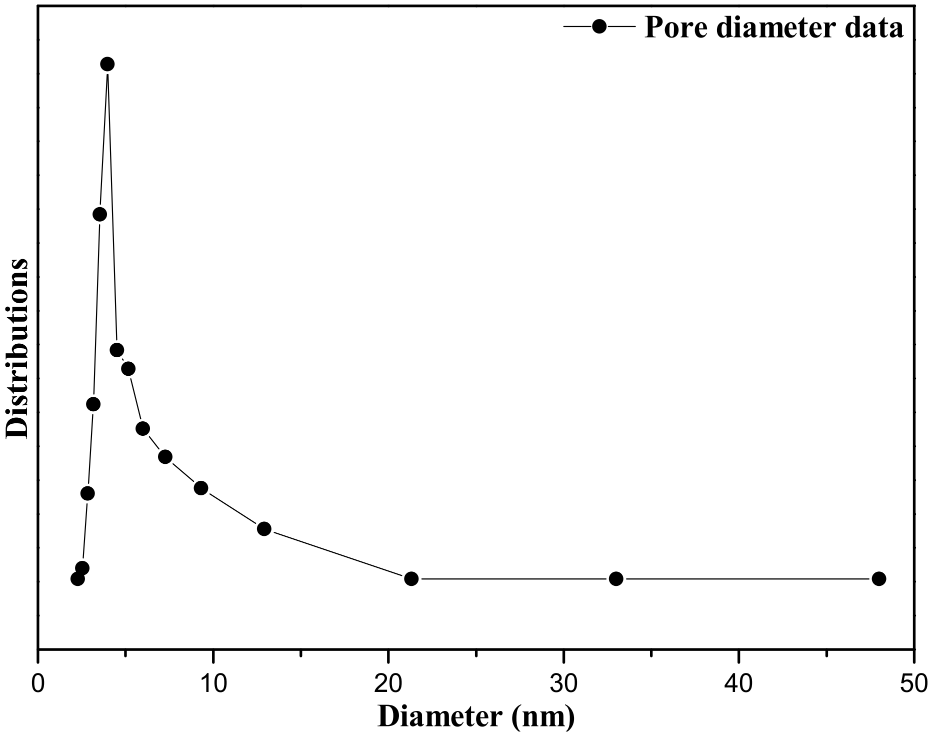

Figure S1. Pore size distributions of the Co-ZIF catalyst.

Table S1 Effects of the mass amount of the Co-ZIF catalyst.

The mass amount of catalyst (g)	Conversion (%)	Selectivity (%)			
		Benzyl alcohol	Benzaldehyde	Benzoic acid	Others
0.16	87.76	12.19	79.76	3.42	4.63
0.18	90.46	8.13	80.18	4.78	6.91
0.20	92.30	3.42	91.31	5.13	0.14
0.22	93.55	2.37	88.75	6.01	2.87
0.24	94.49	1.16	86.49	8.78	3.57

Reaction conditions 0.500 mmol toluene, 20 mL HFIP, 0.004 g NHPI, 0.12 MPa O_2, 313 K and 240 min.

Table S2 Effects of solvent.

Solvent	Conversion (%)	Selectivity (%)			
		Benzyl alcohol	Benzaldehyde	Benzoic acid	Others
acetone	14.35	43.25	21.46	23.57	11.72
cyclohexane	12.34	34.68	13.47	37.65	14.20
trichloromethane	25.46	25.68	31.49	29.42	13.41
ethyl acetate	36.72	31.45	30.27	29.41	8.87
ethanol	62.89	26.77	29.43	32.56	11.24
tetrahydrofuran	57.96	22.38	32.71	26.92	17.99
DMF	63.12	30.46	35.46	24.59	9.49
isopropanol	82.35	12.92	61.63	15.51	9.94
HFIP	92.30	3.42	91.31	5.13	0.14

Reaction conditions 0.20 g Co-ZIF, 0.500 mmol toluene, 20 mL HFIP, 0.004 g NHPI, 0.12 MPa O_2 , 313 K and 240 min.
